# Personalized Clinical Decision Making Through Implementation of a Molecular Tumor Board: A German Single-Center Experience

**DOI:** 10.1200/PO.18.00105

**Published:** 2018-08-16

**Authors:** Rouven Hoefflin, Anna-Lena Geißler, Ralph Fritsch, Rainer Claus, Julius Wehrle, Patrick Metzger, Meike Reiser, Leman Mehmed, Lisa Fauth, Dieter Henrik Heiland, Thalia Erbes, Friedrich Stock, Agnes Csanadi, Cornelius Miething, Britta Weddeling, Frank Meiss, Dagmar von Bubnoff, Christine Dierks, Isabell Ge, Volker Brass, Steffen Heeg, Henning Schäfer, Martin Boeker, Justyna Rawluk, Elke Maria Botzenhart, Gian Kayser, Simone Hettmer, Hauke Busch, Christoph Peters, Martin Werner, Justus Duyster, Tilman Brummer, Melanie Boerries, Silke Lassmann, Nikolas von Bubnoff

**Affiliations:** **All authors:** University of Freiburg, Freiburg; **Ralph Fritsch**, **Julius Wehrle**, **Cornelius Miething**, **Christoph Peters**, **Martin Werner**, **Justus Duyster**, **Tilman Brummer**, **Melanie Boerries**, **Silke Lassmann**, and **Nikolas von Bubnoff**, German Cancer Consortium, partner site Freiburg, and German Cancer Research Center, Heidelberg; **Rainer Claus**, Augsburg Medical Center, Augsburg; and **Hauke Busch**, University of Lübeck, Lübeck, Germany.

## Abstract

**Purpose:**

Dramatic advances in our understanding of the molecular pathophysiology of cancer, along with a rapidly expanding portfolio of molecular targeted drugs, have led to a paradigm shift toward personalized, biomarker-driven cancer treatment. Here, we report the 2-year experience of the Comprehensive Cancer Center Freiburg Molecular Tumor Board (MTB), one of the first interdisciplinary molecular tumor conferences established in Europe. The role of the MTB is to recommend personalized therapy for patients with cancer beyond standard-of-care treatment.

**Methods:**

This retrospective case series includes 198 patients discussed from March 2015 through February 2017. The MTB guided individual molecular diagnostics, assessed evidence of actionability of molecular alterations, and provided therapy recommendations, including approved and off-label treatments as well as available matched clinical trials.

**Results:**

The majority of patients had metastatic solid tumors (73.7%), mostly progressive (77.3%) after a mean of 2.0 lines of standard treatment. Diagnostic recommendations resulted in 867 molecular diagnostic tests for 172 patients (five per case), including exome analysis in 36 cases (18.2%). With a median turnaround time of 28 days, treatment recommendations were given to 104 patients (52.5%). These included single-agent targeted therapies (42.3%), checkpoint inhibitors (37.5%), and combination therapies (18.3%). Treatment recommendations were implemented in 33 of 104 patients (31.7%), of whom 19 (57.6%) showed stable disease or partial response, including 14 patients (7.1% of the entire population) receiving off-label treatments.

**Conclusion:**

Personalized extended molecular-guided patient care is effective for a small but clinically meaningful proportion of patients in challenging clinical situations. Limited access to targeted drugs, lack of trials, and submission at late disease stage prevents broader applicability, whereas genome-wide analyses are not a strict requirement for predictive molecular testing.

## INTRODUCTION

Personalized cancer medicine uses molecular biomarkers for standard-of-care treatment stratification, such as activating *BRAF* mutations for the treatment of melanoma with BRAF inhibitors.^1^ In parallel, it has become evident that therapeutic strategies with targeted drugs are no longer specific for the treatment of distinct entities but rather for particular molecular profiles across different cancers.^2-4^ Thus, testing for single-drug targets can provide therapeutic information, but its predictive value may vary between entities. Although an activating *BRAF* V600E mutation will predict response to BRAF inhibitors in melanoma,^1^ it may not do so in colorectal cancers because of epidermal growth factor receptor (EGFR) feedback activation with requirement of additional EGFR targeting.^5,6^ Moreover, non-V600 *BRAF* mutations might not be responsive to BRAF inhibition at all.^7^ Thus, one-mutation–one-drug approaches may be ineffective, especially in heavily pretreated patients with cancer. Underlying causes include the challenge to discriminate relevant mutations and pathway aberrations from background and passenger mutations,^8^ the clonal molecular intra- and intertumoral heterogeneity,^9,10^ and dynamic changes in the molecular composition of cancer, especially if treatment leads to selection of resistant subclones. Examples include the selection of *RAS* mutant clones in colorectal cancer treated with EGFR antibodies, such as cetuximab or panitumumab,^11^ or the acquisition of a secondary *EGFR* T790M kinase domain mutation mediating resistance to EGFR kinase inhibitors, such as gefitinib or erlotinib in non–small-cell lung cancer.^12,13^

This increasing amount of complexity requires tools to translate individual information into personalized treatment concepts. A molecular tumor board (MTB) represents a platform that integrates clinical and molecular parameters for clinical decision making. Here, we report the 2-year experience of the Comprehensive Cancer Center Freiburg MTB that provides personalized treatment recommendations on the basis of individual molecular diagnostics. We hereby present detailed data on patient characteristics, treatment recommendations, clinical adherence to recommendations, and outcomes of treated patients.

## METHODS

### MTB Implementation and Organization

**Fig A1.**
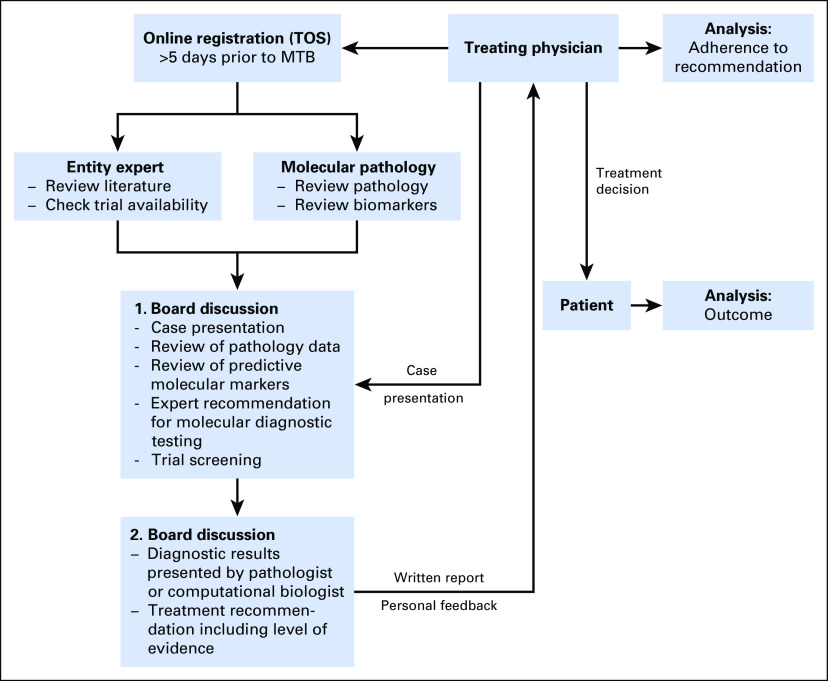
Molecular Tumor Board (MTB) workflow. TOS, Tumorboard Online System.

The MTB is run by an interdisciplinary team of medical and scientific experts with a focus on clinical and translational oncology and computational and molecular biology. Cases are submitted using an online registration and documentation system (Appendix Fig A1). Each case is assigned to a clinician scientist with expertise in the specific cancer type (entity expert), who reviews the literature and available clinical trials. In parallel, the molecular pathology team reviews the individual tumor pathology and sets up a presentation of already performed and suggested diagnostic tests. The initial discussion includes a clinical case presentation, review of the pathology data and the tumor-specific genetic landscape, known molecular predictive or prognostic markers, active clinical trials, and potential in- and off-label molecular targeted treatments. The molecular diagnostic requests are performed using certified and standard operating procedure (SOP)–driven processes. Diagnostic results are presented to the MTB by the molecular pathology and/or the computational biologist team. After discussion, treatment recommendations are given and are supported by levels of evidence (Data Supplement). These are based on published molecular biomarker recommendations.^14^

### Patients and Patient Informed Consent

All patients discussed (n = 198) were included in this retrospective single-center case series. All molecular diagnostic tests were conducted in accordance with the medical treatment contract signed by each patient. Patient tissue was stored in the local biobank and required a signed informed consent, approved by the University of Freiburg institutional review board. Patients with individual or family history indicative of germline disease-causing mutations were referred to the Institute of Human Genetics for counseling and possibly germline genetic analyses.

### Diagnostic Molecular Pathology

**Fig 1.**
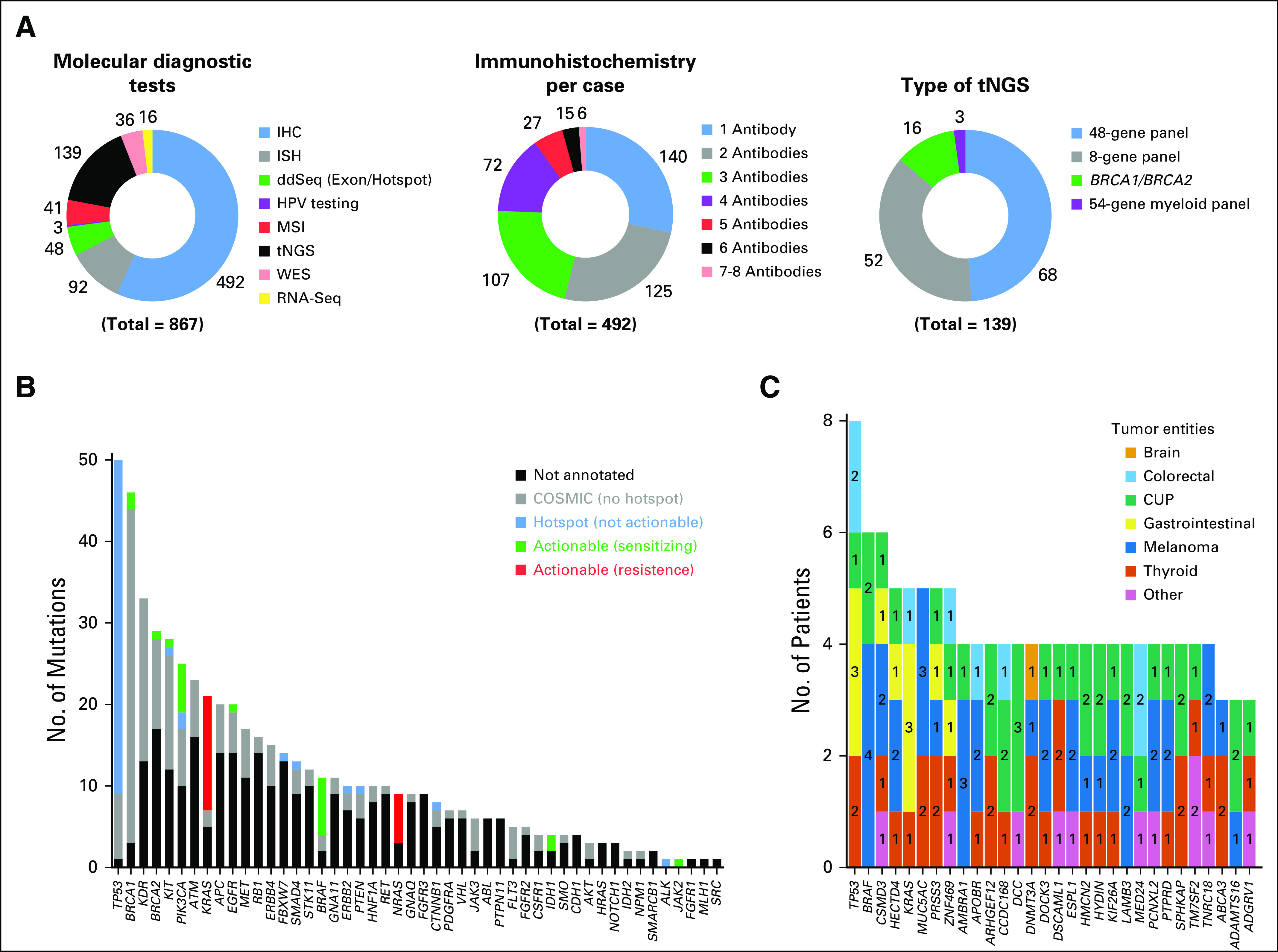
Molecular diagnostic testing. (A) The panels depict the type of molecular diagnostic testing performed (left panel) and specify the number of immunohistochemical stains (one to eight antibodies) per case (middle panel) as well as the type of targeted next-generation sequencing (tNGS) library sequenced (right panel). tNGS was performed either by a custom panel (eight-gene panel), a 48-gene panel (TruSeq Amplicon Cancer Panel, Illumina, San Diego, CA), a 54-gene myeloid panel (TruSight Myeloid Sequencing Panel, Illumina) or a custom *BRCA1/2* consortium panel. (B) The bar plot depicts the number of sequence variants detected in tumor DNA of 139 patients using tNGS. The bars indicate the numbers of mutations in a given gene (black) and sequence variants that are annotated in COSMIC (gray). The numbers of actionable mutations is shown in green (drug sensitizing) and red (drug resistance). (C) The bar plot depicts the 30 most frequently somatic mutated genes of 36 patients analyzed by whole-exome sequencing (WES). The colors indicate different tumor entities. Mutations with a variant allele frequency > 10% and a minor allele frequency < 0.001 were considered. The GI tumor category includes liver, pancreas, stomach, and esophagus. CUP, carcinoma of unknown primary; HPV, human papillomavirus; IHC, immunohistochemistry; ISH, in situ hybridization; MSI, microsatellite instability.

Appropriate tissues were subjected to molecular analyses as recommended by the MTB (Fig 1). All analyses were carried out according to routine pathology laboratory testing procedures, with assays being nationally validated and certified. Targeted next-generation sequencing (tNGS) included a custom-designed hotspot eight-gene panel (designed by S.L. and produced by Illumina, San Diego, CA), a *BRCA1/2* panel (produced by Illumina), a hotspot 48-gene panel (TruSeq Amplicon Cancer Panel, Illumina), and a 54-gene myeloid panel (TruSight Myeloid Sequencing Panel, Illumina).^15-17^

### Investigational Genetic Tumor Characterization

Whole-exome sequencing (WES) and RNA sequencing (RNA-Seq) were performed on tumor tissue. Complementary germline DNA was obtained from peripheral blood or healthy tissue. Only nonsynonymous mutations detected with a variant allele frequency > 10% and listed with a minor allele frequency < 0.001% by the Exome Aggregation Consortium^18^ were reported. Single nucleotide variations were classified according to ClinVar,^19^ COSMIC,^20^ dbSNP,^21,22^ hotspot mutation^23,24^ (http://cancerhotspots.org/#/home), TARGET db (http://archive.broadinstitute.org/cancer/cga/target), drug-gene interaction (DGIdb; http://www.dgidb.org),^25^ and CADD (http://cadd.gs.washington.edu), and categorized according to the predicted impact on protein function by Condel.^26-31^ Copy number alteration analysis was performed using Control-FREEC.^32^ The STAR^33^ aligner was used to align and infer the gene expression level. FusionCatcher (https://doi.org/10.1101/011650) was used to predict gene fusions. Differentially expressed genes were identified using the limma-voom package from R/Bioconductor.^34,35^

## RESULTS

From March 2015 through February 2017, 49 MTB meetings were attended by a median of 16 physicians and scientists, ensuring continuous interdisciplinary data interpretation and discussions with diagnostic and therapeutic decision making. The workflow of the MTB included a case and literature review, molecular diagnostic recommendations, and follow-up discussions of the molecular diagnostic results, including treatment recommendations (Appendix Fig A1). Thus, a total of 385 case discussions were held for 198 patients (1.9 discussions per patient; Table 1). In total, 505 structured recommendations were given (2.5 per patient; Table 1). These included 305 diagnostic and 104 treatment recommendations.

**Table 1.**
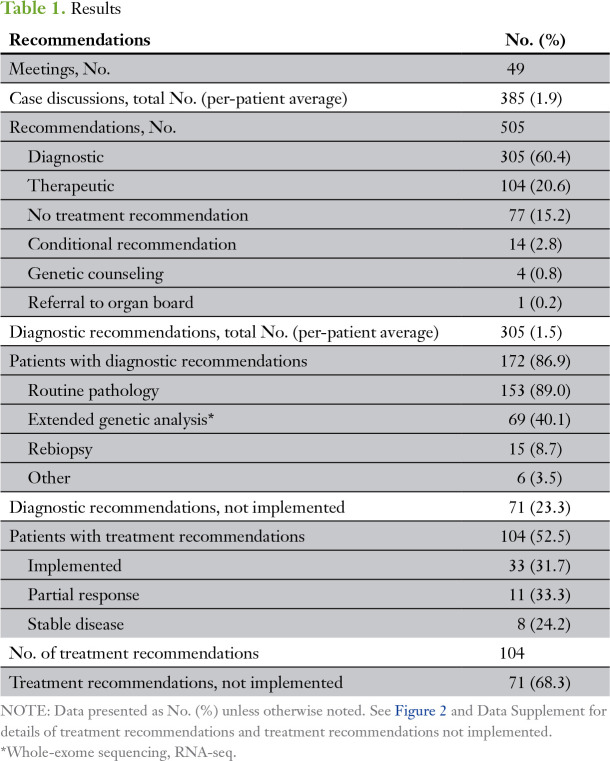
Results

### Patient Characteristics

The average patient age at the time of the initial MTB presentation was 58 years (range, 1 to 85 years). Detailed patient characteristics are listed in Table 2. One hundred ninety-one of 198 patients (96.4%) had an underlying malignant condition. Patients with solid tumors largely outbalanced hematopoietic malignancies (95.5% *v* 4.5%). Soft tissue tumors (12.6%), CNS tumors (11.1%), and carcinoma of unknown primary (CUP; 10.1%) were the most frequent tumor entities. The majority of patients (n = 146; 73.7%) suffered from metastatic disease, and 77.3% (n = 153) showed disease progression while receiving the standard treatment (Table 2). The mean time interval from diagnosis to first MTB discussion was 33.6 months (range, 1 to 541 months). Patients with treatment-refractory metastatic disease had undergone a mean of 2.0 (range, one to 11) lines of systemic pretreatments. A minority of the patients was referred to the board with rare tumors (n = 33; 16.7%) or because of young age (n = 3; 1.5%).

**Table 2.**
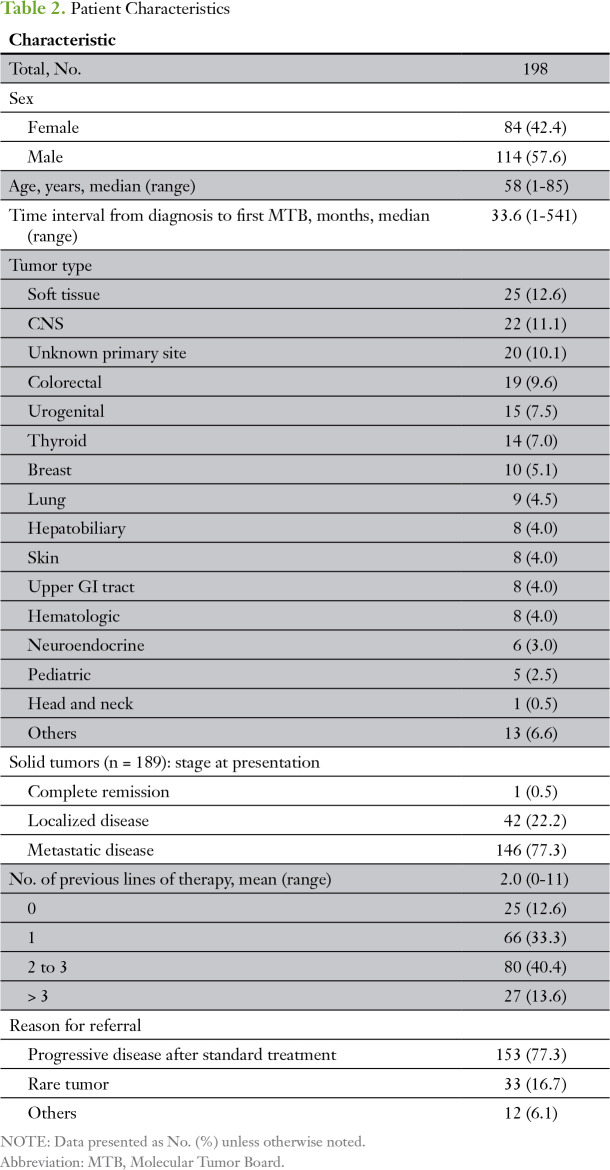
Patient Characteristics

### Molecular Diagnostic Testing

The distribution of molecular diagnostic recommendations is shown in Table 1. For 172 patients (86.9%), 305 recommendations were given and included routine molecular tests in 153 (89%), extended genetic analysis in 69 (40.1%), and both in 53 (30.8%) patients. Rebiopsies were recommended in 15 cases, mostly because of lack of adequate tissue. Of all diagnostic recommendations, 234 (76.7%) were implemented, resulting in 867 single diagnostic tests (mean, five per patient), including 815 routine molecular tests and 52 extended genetic analyses (Fig 1A, left panel).

Routine molecular diagnostics included immunohistochemical (IHC) staining for biomarkers (n = 492; Fig 1A, middle panel), such as programmed death-ligand 1 (PD-L1) and mismatch repair proteins, in situ hybridizations (ISH) for gene copy number analyses (n = 92), and testing for microsatellite instability and/or gene hotspot variations (n = 89) and tNGS (n = 139; Fig 1A). The latter included libraries of different gene panels (Fig 1A; right panel). The most frequent COSMIC annotated sequence variants detected by tNGS occurred in *TP53*, *BRCA1*, *KDR*, *KIT*, *KRAS*, *PIK3CA*, *BRCA2*, and *BRAF* (Fig 1B; Data Supplement). Therapeutically relevant mutations in hotspot regions were identified in 41 of 139 patients (29.5%), including drug-sensitizing variants in *BRAF*, *PIK3CA*, *IDH1*, *EGFR*, and *KIT*, as well as drug resistance variants in *KRAS* and *NRAS*.

Extended genetic analyses including exome and transcriptome assays were performed for 36 patients (18.2%; WES and RNA-Seq: n = 35; RNA-Seq only: n = 1). In those, we identified a total of 5,335 variants, including 18 COSMIC annotated hotspot mutations (Data Supplement). Sixteen were classified as therapy relevant according to the DGI and the TARGET databases. Among the remaining non-hotspot mutations, 1,518 were annotated in COSMIC, including 288 and 28 mutations annotated in DGI and TARGET databases, respectively (Data Supplement). A total of 3,799 mutations were unknown to COSMIC (Data Supplement). The disease impact of non-hotspot mutations is more difficult to evaluate; however, it can lead to additional therapy-relevant insights. For example, the *ERBB2* S656F mutation might, according to TARGET and DGI databases, constitute an activating mutation, therefore targetable by trastuzumab or lapatinib. The most frequently mutated genes were *TP53* and *BRAF* (Fig 1C).

Overall, 71 of 305 diagnostic recommendations (23.3%) were not pursued. As shown in the Data Supplement, reasons for nonadherence included technical reasons (53.5%; mostly lack of sufficient tissue or DNA/RNA), patient death (12.7%), loss to follow-up (11.3%), medical reasons (9.9%), or patient will (9.9%).

### Treatment Recommendations

**Fig 2.**
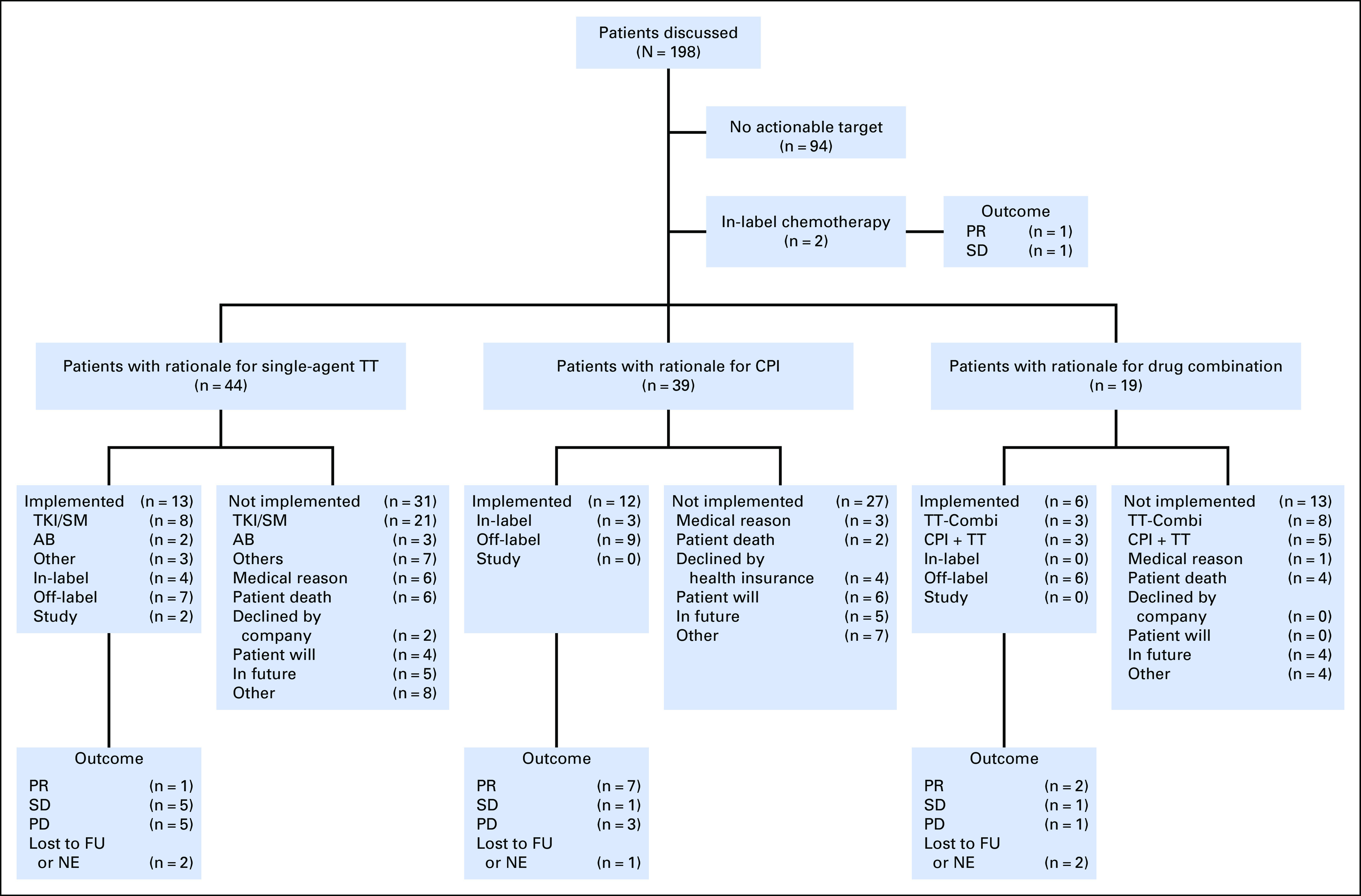
Flow diagram of patients discussed at the Molecular Tumor Board. Responses were determined according to Response Evaluation Criteria in Solid Tumors (RECIST) version 1.1. AB, antibody; Combi, combination; CPI, checkpoint inhibitor; FU, follow-up; NE, not evaluable; PD, progressive disease; PR, partial remission; SD, stable disease; SM, small molecule; TKI, tyrosine kinase inhibitor; TT, targeted therapy.

Specific treatment recommendations were given to 104 patients (Table 1; Fig 2) and mainly included off-label immune checkpoint inhibitor (CPI; n = 36; 34.6%), off-label targeted therapy (n = 19; 18.3%) with tyrosine kinase inhibitors, small molecules or antibodies that were not CPI (AB), trial inclusions (n = 13; 12.5%), and off-label combination treatments (n = 18; 17.3%; Data Supplement; Fig 2). Ninety of 104 treatment recommendations (86.5%) were either off-label therapies (n = 77) or trial inclusions (n = 13).

The implementation rate of treatment recommendations was 31.7% (33 of 104). In-label recommendations were pursued in nine of 14 cases (64.3%), whereas off-label recommendations and trial inclusions were implemented in only 28.6% (22 of 77) and 15.4% (two of 13) of the cases, respectively. Intended trial inclusion in 11 patients failed because of poor performance status or patient death (n = 5), closed trial arm (n = 4), or patient will (n = 2). Main reasons for nonimplementation of treatment recommendations included loss to follow-up (22.5%), recommendation in the future (19.7%), patient death (16.9%), patient will (14.1%), and medical reasons (14.1%; Data Supplement). Of note, evidence level of individual off-label recommendations did not affect implementation rates (data not shown).

### Clinical Outcome

**Fig 3.**
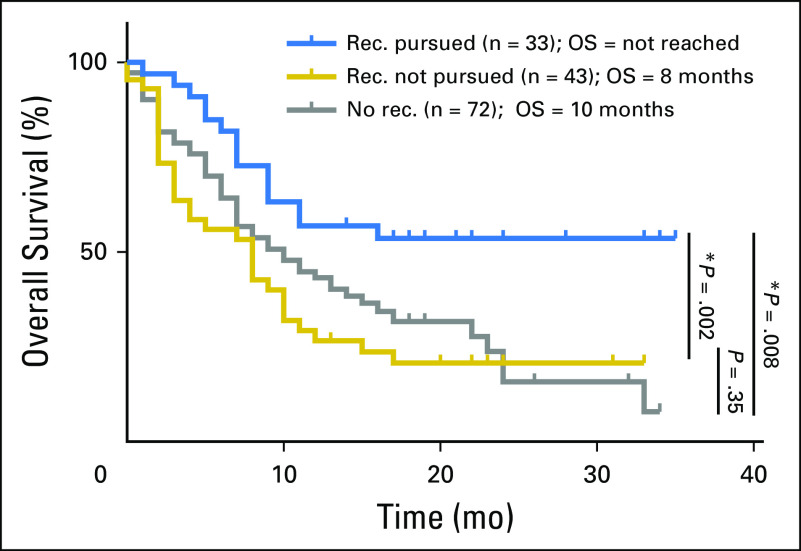
Survival analysis. The Kaplan-Meier curve shows the survival of the following three subgroups of patients with stage IV malignancies (n = 148): patients who implemented the treatment recommendation (Rec. pursued, n = 33), patients who did not implement the treatment recommendation (Rec. not pursued, n = 43; of note: patients who did not receive the recommended therapy because of death before treatment initiation [n = 12] were excluded from analysis), and patients who did not receive a treatment recommendation (n = 72). The curve comparison with the log-rank (Mantel-Cox) test revealed statistical significant differences as shown on graph. OS, overall survival. (*) *P* < .01.

In 33 patients with implemented treatment recommendations, partial remissions (PR) and stable diseases (SD) were seen in 11 (33.3%) and eight patients (24.2%; Table 1), respectively. After excluding in-label therapies, nine patients achieved PR and five patients SD, resulting in an overall response rate of 4.6% (nine of 198 patients) and a disease control rate (DCR) of 7.1% (14 of 198 patients). Of note, all five patients experiencing SD experienced disease progression while receiving the previous treatment. Of 14 responders receiving off-label therapies, eight (57.1%) showed a progression-free survival (PFS) ratio (PFS2/PFS1; PFSr) > 1.3, supporting the impact of the recommended therapies.^36^ Three patients had a PFSr < 1.3 with ongoing responses, meaning that their PFSr is still increasing. Details about the outcome of responding patients are shown in Table 3. Two individual cases are shown in the Data Supplement. Adherence to recommendations and outcome according to type of treatment is shown in Fig 2. To assess whether implementation of treatment recommendations affected overall survival from first MTB discussion, we analyzed all patients with stage IV malignancies according to three subgroups (n = 148; Fig 3). The median survival was not reached for patients with implemented treatment recommendations (n = 33 recommendations pursued; 95% CI, 9 months to not reached), 8 months for patients for whom treatment recommendations were not implemented (n = 43 recommendations not pursued; 95% CI, 3 to 10 months), and 10 months for patients who did not receive a treatment recommendation (n = 72 no recommendations; 95% CI, 7 to 17 months). Patients who did not receive the recommended therapy because of death before treatment initiation (n = 12) were excluded from analysis.

**Table 3.**
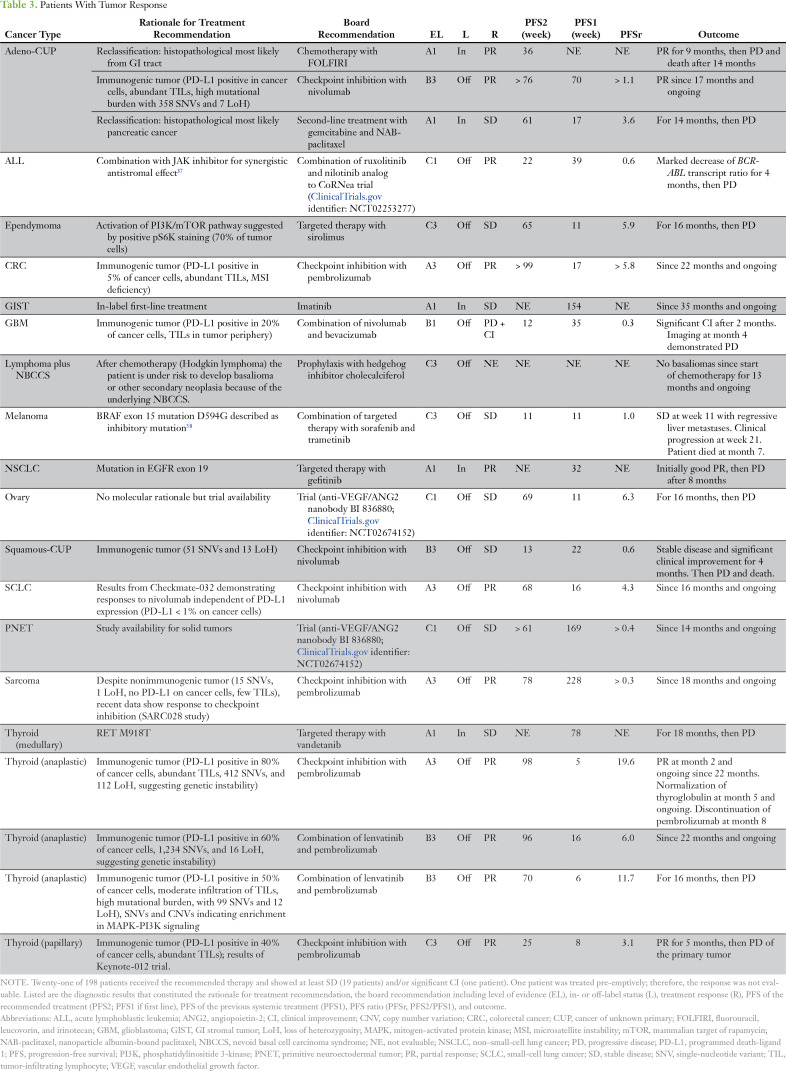
Patients With Tumor Response

## DISCUSSION

In a cohort of 198 patients with mostly advanced malignancies beyond standard-of-care treatment, the Comprehensive Cancer Center Freiburg MTB identified actionable targets in 52.5% of the cases. Thirty-two percent received the recommended treatment. In 33 patients with implemented treatment recommendations the disease-control rate was 57.6%; it was 9.6% (19 of 198 patients) for the entire cohort. Because the primary goal of an MTB is to give treatment recommendations beyond standard of care, we excluded five responders who received in-label therapies resulting in a DCR of 7.1% (14 of 198 patients). Other MTB case series reported DCRs in 3.2%, 7.8%, 9%, and 23.3% of the patients,^38-41^ suggesting that approximately 10% of patients might benefit from advanced personalized decision making.

Although molecular heterogeneity will limit the effect of therapeutic kinase inhibitors, higher nonsynonymous mutational burden can create more neoantigens and therefore improve response rates to CPI.^42,43^ In our series, eight of 11 patients (72.7%) showing PR received CPI, including seven off-label uses. Predictive biomarkers for individualized immunotherapies are emerging and changing rapidly, with strong differences between entities.^44^ Here, we used IHC for programmed cell death protein 1 (PD-1)/PD-L1, tumor-infiltrating lymphocytes, microsatellite instability testing, and mutational burden assessment as predictive biomarkers. In the near future, identifying individual cancer neoantigens might allow a more precise prediction of responses to immunotherapies.^45^ This highlights the importance of an interdisciplinary MTB team that analyzes and interprets biomarkers to identify patients who might benefit from off-label immuno-oncology treatments.

In an MTB workflow, the portfolio of molecular diagnostic tests, as well as criteria to match and prioritize targeted therapies to molecular biomarkers, affects the probability to identify patients with actionable targets. Here, we used customized molecular diagnostics, including IHC/ISH and tNGS, in 153 out of 198 patients (77.3%) We implemented WES or RNA-Seq analyses for patients with carcinomas of unknown primary and rare cancers and with diseases in which routine molecular diagnostics did not reveal any actionable target (18.2% of patients).

Multidimensional data have not been implemented successfully to clinical routine, partly because of the complexity of developing and evaluating mathematical predictive models.^46,47^ A recent analysis showed that an MTB workflow including WES/whole-genome sequencing, RNA-Seq, and data interpretation by a multidisciplinary board required a turnaround time of 6 weeks.^48^ Using high-dimensional molecular data, the Molecular Screening for Cancer Treatment Optimization (MOSCATO-01) trial reported actionable mutations in less than half of the patients with advanced solid tumors,^49^ and in the National Cancer Institute Molecular Analysis for Therapy Choice (NCI-MATCH) trial, only 9% of the patients could be assigned to one of the prespecified treatment arms.^50^ In contrast, our approach of customized molecular diagnostic testing with restricted use of extended genetic analyses (WES, RNA-Seq) allows a faster turnover with comparable rates of genetically matched treatment recommendations. Therefore, average costs per case can be reduced at least by half when compared with performing extended molecular analysis for each patient. We identified actionable targets in 52.5% of cases and provided treatment recommendations with a median turnaround time of 28 days. To improve standardization and turnaround time, we recently implemented SOPs for diagnostic work-ups (Data Supplement). Our approach shares similarities with Memorial Sloan Kettering-Integrated Mutation Profiling of Actionable Cancer Targets (MSK-IMPACT), focusing on therapeutically targetable biomarkers for fast clinical decision making and referral of patients to available clinical trials.^51^

Targeted drug combinations might offer better DCR over single-agent therapies.^52-55^ In part, this is due to crosstalk between signaling pathways as well as spatial and temporal clonal heterogeneity, especially in patients with advanced cancer who failed standard-of-care treatment.^56,57^ Most current programs for precision oncology use prespecified, genetically matched, single-agent treatments (NCI-MATCH, ClinicalTrials.gov identifier: NCT02465060; or Targeted Agent and Profiling Utilization Registry [TAPUR], ClinicalTrials.gov identifier: NCT02693535). In our series, three of 19 treatments that successfully controlled disease (15.8%) included molecular combination treatments (Fig 2). These patients did not suffer from grade 3 to 4 adverse effects, although treatment combinations may bear a higher risk of toxicity.^58^

Earlier referral to an MTB (eg, after failure of first-line treatment) might prevent the institution of ineffective treatments, improve the implementation rate, and increase the likelihood of success of molecular biomarker–matched treatments. In our series, patient death, patient preference, or medical reasons precluded implementation in 23.3% of diagnostic and 68.3% of treatment recommendations. The survival analysis revealed a significant overall survival advantage for patients with implemented MTB treatment recommendations (median overall survival not reached; 95% CI, 9 months to not reached) compared with patients where recommendations were not pursued (8 months; 95% CI, 3 to 10 months; *P* = .002) as well as for patients without treatment recommendation (10 months; 95% CI, 7 to 17 months; *P* = .008). Because of the low sample size and the heterogeneous composition of patients in the cohorts, the validity of this survival analysis is limited.

Access to molecular biomarker–matched, off-label agents for cancer treatment is limited. In a recent single-center study, only 5% of molecular biomarker–matched treatment recommendations were implemented, mainly because of limited access to clinical trials or to restricted use of drugs outside their marketed label.^59^ Thus, it is crucial to build up platforms for patients and treating physicians to link individual molecular information of the tumor to appropriate nonapproved drugs and available clinical trials. To this end, MTB networks might implement SOPs for diagnostic work-ups and data interpretation and build alliances to governmental institutions and insurance companies to generate criteria for the financial coverage of molecular analyses and off-label treatments. Finally, an MTB is predestined to generate knowledge and evidence in oncology via single-person trials instead of large, time- and cost-intensive clinical trials. In case of sequence variants with undetermined significance, precision oncology workflows should allow fast reverse translation of sequence variants into informative preclinical models. In a patient with melanoma, we identified a kinase-inactivating *BRAF* mutation (Data Supplement). In vitro characterization indicated antitumor activity of combined pan-RAF and mitogen-activated protein kinase kinase inhibition and guided successful treatment with sorafenib and trametinib. In rare entities, and especially in the setting of treatment-refractory cancers, precision oncology networks should allow hypothesis-driven in vitro studies and validation in small sets of individuals. Thus, within the concept of patient-centric, biomarker-driven trial designs,^60^ an MTB might constitute a critical tool to identify informative patients for clinical trials of targeted therapies in rare molecular subgroups.

In summary, this MTB experience illustrates that patient management, on the basis of individual molecular biomarker profiling and analysis, is feasible in patients beyond standard-of-care treatment. We show a high proportion of trial- and off-label treatment recommendations (86.5%) and a DCR for off-label treatments of 7.1%. In cases where no approved treatment is available, an MTB might allow molecular biomarker–matched off-label use of approved drugs across entity barriers or alternatively facilitate the access to therapeutic basket trials.
